# High-Precision Multi-Object Tracking in Satellite Videos via Pixel-Wise Adaptive Feature Enhancement

**DOI:** 10.3390/s24196489

**Published:** 2024-10-09

**Authors:** Gang Wan, Zhijuan Su, Yitian Wu, Ningbo Guo, Dianwei Cong, Zhanji Wei, Wei Liu, Guoping Wang

**Affiliations:** 1 School of Space Information, Space Engineering University, Beijing 101407, China; dsddfff@stu.cuz.edu.cn (G.W.); ytwu@whu.edu.cn (Y.W.); sxguonb@163.com (N.G.); congdianwei@sina.com (D.C.); weizhanji@163.com (Z.W.); ggfh@stu.cuz.edu.cn (W.L.); m18519338782@163.com (G.W.); 2 State Key Laboratory of Geo-Information Engineering, Xi’an 710054, China

**Keywords:** satellite video, transformer attention learning, multi-object tracking (MOT)

## Abstract

In this paper, we focus on the multi-target tracking (MOT) task in satellite videos. To achieve efficient and accurate tracking, we propose a transformer-distillation-based end-to-end joint detection and tracking (JDT) method. Specifically, (1) considering that targets in satellite videos usually have small scales and are shot from a bird’s-eye view, we propose a pixel-wise transformer-based feature distillation module through which useful object representations are learned via pixel-wise distillation using a strong teacher detection network; (2) targets in satellite videos, such as airplanes, ships, and vehicles, usually have similar appearances, so we propose a temperature-controllable key feature learning objective function, and by highlighting the learning of similar features during distilling, the tracking accuracy for such objects can be further improved; (3) we propose a method that is based on an end-to-end network but simultaneously learns from a highly precise teacher network and tracking head during training so that the tracking accuracy of the end-to-end network can be improved via distillation without compromising efficiency. The experimental results on three recently released publicly available datasets demonstrated the superior performance of the proposed method for satellite videos. The proposed method achieved over 90% overall tracking performance on the AIR-MOT dataset.

## 1. Introduction

It is of great importance to process remote sensing images, especially for Earth observation systems [1,2,3,4,5,6]. In recent years, with the development of remote sensing technology, satellite videos have emerged as a new type of remote sensing data. The interpretation of satellite videos has important applications in commercial monitoring, military surveillance, natural disaster prediction, and so on [7,8,9,10]. This paper aims to tackle the multi-object tracking (MOT) task for satellite videos. The MOT task refers to detecting multiple objects in a video and assigning unique IDs for objects across frames without knowing the number of objects in advance. Recently, researchers have proposed many MOT methods. The most widely used of these methods can be divided into two categories: detection-by-tracking (DBT) [11,12,13,14,15,16] and joint detection and tracking (JDT) [17,18,19,20,21,22].

DBT methods first adopt a object detector to generate object detection bounding boxes in each frame via object detection methods, such as Faster R-CNN [23], SDP [24], DPM [25], etc., and then use a data association approach to associate the detection results and obtain the trajectories. SORT [11] is the most classic DBT method, which is mainly composed of object detection, a Kalman filter [26], and the Hungarian algorithm [27]. DeepSORT [13] improves SORT by introducing a deep learning model to extract appearance features for closest proximity matching during real-time tracking. Then, StrongSORT [15] further improves the method in various aspects of object detection, feature representation, and matching. In addition, ByteTrack [12], GHOST [28], IOU-Tracker [16], and so on have also been proposed and follow the DBT framework. DBT-based methods usually have high tracking accuracy, which benefits from specialized and separate detection and data association methods. However, using these two separate steps results in relatively low tracking efficiency.

JDT methods achieve the MOT task via multi-task learning in an end-to-end network, namely by simultaneously accomplishing object detection and data association tasks. CenterTrack [19], which is based on CenterNet [29], inputs two consecutive frames and a heat map of the previous frame and directly outputs the location, size, confidence score, and ID of targets. The goal is to detect and track objects simultaneously via a shared feature learning network. Based on CenterNet, FairMOT [21] estimates target centers and positions on high-resolution feature maps and introduces parallel branches to estimate pixel-level Re-ID features. TraDeS [22] estimates the offsets of tracked targets based on cost amounts. TrackFormer [30] conducts joint object detection and tracking via transformer-based attention that connects past and future frames. With shared networks, JDT methods can usually track objects efficiently. However, due to their use of point-based detection, their tracking results are often less stable and accurate than those of DBT methods. A comparison between DBT and JDT methods is summarized in Table 1.

The DBT and JDT methods have complementary properties. If we use an end-to-end network for tracking while training it with both an object detection network and a tracking head, then object representation can be enhanced without influencing tracking efficiency. As a consequence, in this paper, we propose a transformer distillation-based end-to-end joint detection and tracking framework, which is based on pixel-wise feature distillation and employs a highly precise object detection network as a teacher. In this way, based on an end-to-end network, distilled features are expected to improve the performance of object localization by enhancing key object features. Meanwhile, since the distillation is only implemented in the training process, the tracking efficiency of the tracking network will not be influenced. Considering that targets in satellite videos usually have small scales and are shot from a bird’s-eye view, we propose a pixel-wise transformer-based feature distillation module in order to distill more detailed features from the strong teacher network. Moreover, to distill critical features, we propose a temperature-controllable key feature learning objective function.

As a consequence, based on the principle of improving detection capability of the end-to-end tracking network via distillation without influencing the tracking efficiency, we propose a new transformer distillation-based JDT method and design a corresponding distillation method. During training, it minimizes not only the tracking losses, but also the proposed feature distillation loss. The experimental results demonstrated the effectiveness and efficiency of the proposed method, which could significantly surpass the current state-of-the-art methods.

In general, the contributions of this paper are mainly reflected in the following four aspects:

(1) To meet both the efficiency and accuracy requirements of applications in satellite videos, we propose a transformer distillation-based end-to-end joint detection and tracking (JDT) method for the MOT task. It inherits the efficiency of a JDT-based framework in an end-to-end network during tracking. Meanwhile, it also learns effective object representation from a detection network via a DBT-based framework.

(2) Considering that targets in satellite videos usually have small scales and are shot from a bird’s-eye view, we propose a pixel-wise transformer distillation module to improve tracking accuracy by distilling pixel-wise detail features from a strong teacher network.

(3) Considering that targets in satellite videos usually have similar appearances, we propose a temperature-controllable feature distillation objective function to further enhance the distillation of the useful and critical features of similar targets.

(4) Experimental results on three publicly available datasets (AIR-MOT, SAT-MTB, and VISO) showed that the performance of the proposed method is superior to the baseline method and significantly outperforms the current state-of-the-art methods.

## 2. Related Work

### 2.1. MOT in Satellite Videos

Due to the significance of MOT in a range of applications, many meaningful MOT methods for satellite videos have recently been proposed. Unlike those in nature videos, the targets in satellite videos are usually small and have indistinguishable features. Therefore, motion information is especially important for MOT. In [31], a dataset named VISO was constructed, which mainly focuses on the detection of moving objects in MOT. Moreover, a motion modeling method and a novel detection method were also proposed in [31]. The motion modeling method aims to integrate motion information in MOT by accumulative multi-frame differencing and matrix completion, while the detection method works by modeling local noise via exponential probability distribution with a multi-morphological cue. Focusing on tracking vehicles that are crowded and have similar appearances, a DBT method was proposed in [32] that integrates complementary information between adjacent frames via a customized cross-frame object detection network and captures motion information via a two-branch architecture based on long short-term memory. Detection performance significantly influences the tracking accuracy of MOT. In [33], based on a single-object tracking method, a bidirectional framework was proposed that reduces the degradation of detection networks. Moreover, SFMFMOT, which was proposed in [34], fully considers low-confidence bounding boxes, missed objects, false alarms, and ID switches by exploring slow and motion features. For satellite-based applications, efficiency is also important. A JDT method named TGraM was proposed in [10], which approaches MOT as a graph reasoning procedure and uses a multi-task learning framework. In this paper, considering both the efficiency and accuracy requirements of MOT in satellite videos, we propose a transformer distillation-based end-to-end joint detection and tracking method that learns accurate detection from a teacher network that is based on a JDT baseline.

### 2.2. Transformer-Based MOT Methods

A transformer [35] is a type of neural network that learns contexts and meanings by tracking relationships in sequence data. In recent years, researchers have proposed many multi-object tracking methods based on transformers. GTR [36] takes a short sequence of frames as input and produces global trajectories for all objects. MeMOT [37] employs an online tracking algorithm that performs object detection and data association under a common framework, which is capable of linking objects after a long timespan. MOTR [38] extends DETR [39] and introduces track queries to model the tracked instances in an entire video. It also uses a tracklet-aware label assignment method to train and track newborn object queries, a temporal aggregation network, and a collective average loss to enhance temporal relation modeling. MOTRv2 [40] improves MOTR by elegantly incorporating an extra object detector. P3AFormer [41] tracks objects as pixel-wise distributions. TrackFormer [30] formulates the MOT task as a frame-to-frame set prediction problem based on a transformer. In this paper, to effectively distill object features in satellite videos, we propose a pixel-wise transformer distillation module for accurate MOT in satellite videos.

### 2.3. Knowledge Distillation for MOT

Knowledge distillation [42] is a popular technique that is widely used in many learning tasks, such as facial recognition [43], semantic segmentation [44], behavior prediction [45], and tracking, including single-object tracking [46] and MOT [47]. Knowledge distillation trains a student network not only from data but also from highly precise teacher networks, thereby enhancing the performance of the student network. For MOT, the method proposed in [47] focuses on association performance and distills Re-ID information.

In this paper, we focus on the detection performance of a JDT-based method. The proposed method is different from existing knowledge-distillation-based MOT methods in two ways: first, we propose a transformer-based pixel-wise distillation module, which benefits from learning the features of small targets in satellite videos; second, based on the features of transformer distillation, we propose a temperature-controllable key feature learning objective function, which further improves the feature learning performance of similar targets and tracking performance.

## 3. The Proposed Method

In this study, in order to illustrate the proposed method, we chose a classic, state-of-the-art end-to-end tracking network named CenterTrack [19] as the baseline method. The teacher model we chose was a pre-trained, highly precise object detection network named YOLOX. On this basis, we designed a pixel-wise transformer distillation module to distill object features from YOLOX. Then, to better learn the key features of similar objects, we designed a temperature-controllable key feature learning objective function. In the following sections, we first briefly review CenterTrack, the pixel-wise transformer distillation module, the temperature-controllable key feature learning objective function, and the corresponding optimization derivatives and tracklet generation.

### 3.1. CenterTrack

CenterTrack [19] is an end-to-end JDT framework. The inputs for the network are the current frame $I^{\left(t\right)}\in R^{H\times W\times 3}$, the last frame $I^{\left(t-1\right)}\in R^{H\times W\times 3}$, and the tracked objects in the last frame $T^{\left(t-1\right)}=\left\{b_{0}^{\left(t-1\right)},b_{1}^{\left(t-1\right)},\ldots \right\}$, which are represented in a heat map. During tracking, at frame *t*, the objects detected by CenterTrack are denoted by $T^{\left(t\right)}=\left\{b_{0}^{\left(t\right)},b_{1}^{\left(t\right)},\ldots \right\}$, and their attributes are denoted by b=(p,s,w,id), where $p\in R^{2}$ is the center location, $s\in R^{2}$ represents size, w∈[0,1] reflects detection confidence, and id∈I is the unique identity. To train the network, the overall objective $L^{T}$ should be minimized, including heat map loss, which highlights the center location of targets using the following formula:

(1) $$L_{H}=\frac{1}{N}\left\{\begin{array}{cc} {\left(1-\hat{Y}\right)}^{2}\log\left(\hat{Y}\right) & Y=1\\ {\left(1-Y\right)}^{4}{\hat{Y}}^{2}\log\left(1-\hat{Y}\right) & otherwise \end{array}\right.$$

where *Y* and $\hat{Y}$ denote the predicted and ground-truth heat maps with *N* objects, respectively. Size prediction loss is calculated as follows:

(2) $$L_{S}=\left|Size\left(p\right)-s\right|$$

The offset and center location loss are calculated as follows:

(3) $$L_{O}=\left|\left({\hat{p}}^{t-1}-{\hat{p}}^{t}\right)-\left(p^{t-1}-p^{t}\right)\right|$$

However, in satellite videos, the targets are usually small and have highly similar appearances, which results in confused object localization. As a consequence, in this paper, we propose a pixel-wise transformer distillation module and a temperature-controlled key feature learning objective function to distill object information and increase tracking accuracy in a JDT-based framework while maintaining high tracking efficiency. In this study, CenterTrack was used as the baseline, and we used knowledge distillation to distill detection knowledge from YOLOX into CenterTrack.

### 3.2. Overall Architecture

**Figure 1:**
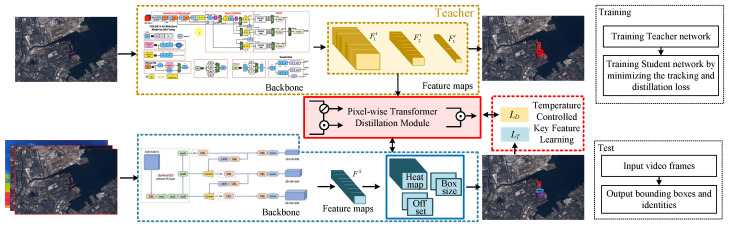
The overall pipeline and flowchart of the proposed method.

The tracking pipeline of the proposed method is shown in Figure 1. The overall framework is composed of a teacher network (a pre-trained object detection network used to distill critical features during training) and a JDT-based student network, which is used for generating ground boxes and trajectories during tracking. The flowchart of training and test are also illustrated. For the t − frame, the inputs of CenterTrack are $I^{\left(t\right)}$, $I^{\left(t-1\right)}$, and $T^{\left(t-1\right)}$, and the feature map $F^{s}$ is obtained through the backbone network $\phi_{T}$ as follows:

(4) $$F^{s}=\phi_{T}\left(I^{\left(t\right)}\right)$$

At the same time, the feature maps $\left\{F_{1}^{t},F_{2}^{t},F_{3}^{3}\right\}$ of image $I^{\left(t\right)}$ are obtained through the object detection backbone network $\phi_{D}$ as follows:

(5) $$\left\{F_{1}^{t},F_{2}^{t},F_{3}^{3}\right\}=\phi_{D}\left(I^{\left(t\right)}\right)$$

We regard the features output by the object detection network as *Key* (K) features and the features obtained by the tracking network as *Query* (Q) features. Since targets in satellite videos are usually small and taken from a bird’s-eye view, we use transformer-based pixel-wise attention to distill *Key* features. Considering that targets in satellite videos also usually have similar appearances, we added a temperature-controllable key-feature-learning-based objective function $L_{D}$ to $L_{T}$, as in CenterTrack, for optimization to obtain the final results. The pixel-wise transformer distillation and temperature-controlled key feature learning objective are introduced in the following sections.

### 3.3. Pixel-Wise Transformer Distillation

**Figure 2:**
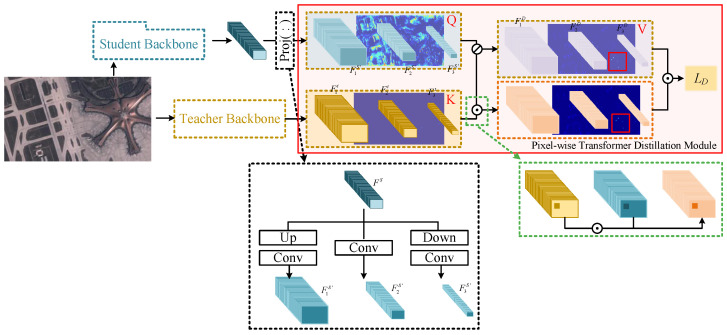
Pixel-wise transformer distillation.

To distill the object information in the baseline, i.e., CenterTrack, we designed a pixel-wise transformer distillation architecture, as shown in Figure 2. Before distillation learning, a highly precise teacher detection network $\phi_{D}$ is first pre-trained, as shown in Figure 1. Then, the multi-scale features $F^{t}$ extracted from the network can effectively represent the semantic features of the corresponding targets. As a consequence, if these features can be well distilled in the JDT-based tracking network, its detection performance is expected to improve without impacting tracking efficiency.

During training, with the training frame $I^{\left(t\right)}$, the highly precise object features $F_{1}^{t}$, $F_{2}^{t}$, and $F_{3}^{t}$ at different scales are first extracted via $\phi_{D}$. Meanwhile, $I^{\left(t\right)}$ is fed into the CenterTrack network $\phi_{T}$, and the tracking feature $F^{s}$ is then generated and fed into two branches: one is the tracking head to generate tracking loss for optimization; the other is for object feature distillation. To distill multi-scale features, considering the scale differences between $F^{s}$ and $F_{1}^{t}$, $F_{2}^{t}$, and $F_{3}^{t}$, $F^{s}$ is first projected into multi-scale feature maps $F_{1}^{s^{\prime }}$, $F_{2}^{s^{\prime }}$, and $F_{3}^{s^{\prime }}$ via several convolutional layers, as follows:

(6) $$F_{1}^{s^{\prime }}=Conv_{1\times 1}\left(Conv_{3\times 3}\left(Conv_{1\times 1}\left(F^{s}\right)\right)\right)$$

(7) $$F_{2}^{s^{\prime }}=Conv_{1\times 1}\left(Conv_{3\times 3}\left(Conv_{1\times 1}\left(F_{1}^{s^{\prime }}\right)\right)\right)$$

(8) $$F_{3}^{s^{\prime }}=Conv_{1\times 1}\left(Conv_{3\times 3}\left(Conv_{1\times 1}\left(F_{2}^{s^{\prime }}\right)\right)\right)$$

Then, the feature maps of $F_{1}^{D},F_{2}^{D},$ and $F_{3}^{D}$ are constructed as follows:

(9) $$F_{1}^{D}=F_{1}^{t}-F_{1}^{s^{\prime }}$$

(10) $$F_{2}^{D}=F_{2}^{t}-F_{2}^{s^{\prime }}$$

(11) $$F_{3}^{D}=F_{3}^{t}-F_{3}^{s^{\prime }}$$

The aim of distillation is to minimize differences; however, tracking performance may decrease due to irrelevant features from the detection network. As a consequence, we constructed a pixel-wise transformer distillation module.

To distill tracking-related features, the features of *K* and *Q* are first constructed as $F^{t}$ and $F^{s^{\prime }}$, respectively. Then, the final pixel-wise transformer distillation-based difference $F_{i}^{D^{\prime }}$ is generated via transformer-based attention as follows:

(12) $$F_{1}^{D^{\prime }}=Softmax\left(F_{1}^{s^{\prime }}⊙F_{1}^{t}\right)⊙F_{1}^{D}$$

(13) $$F_{2}^{D^{\prime }}=Softmax\left(F_{2}^{s^{\prime }}⊙F_{2}^{t}\right)⊙F_{2}^{D}$$

(14) $$F_{3}^{D^{\prime }}=Softmax\left(F_{3}^{s^{\prime }}⊙F_{3}^{t}\right)⊙F_{3}^{D}$$

where ⊙ denotes the element-wise multiplication operator.

Using the transformer-based module, object features relevant to tracking can be specifically highlighted and distilled. Moreover, the pixel-wise transformer distillation module is implemented at different scales, so the features of both large and small objects can be extracted, and feature distillation effectiveness can be improved. For the pixel-wise transformer distillation module, the distillation objective function is defined based on $F^{D^{\prime }}$.

### 3.4. Temperature-Controlled Key Feature Learning

In contrast to existing feature distillation methods, which usually directly minimize the distance between two features, we aimed to distill critical target features and restrain background features in distillation. As a consequence, we designed a new distillation objective and introduced a distance temperature *T* to control the generalization of distilled features. This objective is used to highlight target information and distill critical features.

Using the tracking loss $L^{T}$ defined in CenterTrack, we developed a temperature-controllable feature distillation loss $L^{D}$ by introducing temperature *T* as follows:

(15) $$L_{1}^{D}=exp\left(F_{1}^{D^{\prime }}⊙F_{1}^{D}/T\right)$$

(16) $$L_{2}^{D}=exp\left(F_{2}^{D^{\prime }}⊙F_{1}^{D}/T\right)$$

(17) $$L_{3}^{D}=exp\left(F_{3}^{D^{\prime }}⊙F_{1}^{D}/T\right)$$

The total distillation loss $L^{D}$ is the sum of $L_{1}^{D}$, $L_{2}^{D}$, and $L_{3}^{D}$:

(18) $$L^{D}=L_{1}^{D}+L_{2}^{D}+L_{3}^{D}$$

Here, *T* controls the variance among different feature values in the feature maps. Lower *T* values result in a larger variance between small and large feature values, which can highlight more critical objects and significantly restrain background objects. Meanwhile, larger *T* values result in lower variance, and background information can also be distilled into the tracking network. With larger *T* values, the feature maps highlight many background objects, while lower *T* values filter out most objects. Larger *T* values focus on more objects, and more targets can be distilled. With this controllable temperature, useful features for tracking can be effectively distilled.

Then, with the tracking loss, the final objective is defined as follows:

(19) $$L=L^{T}+\lambda L^{D}$$

where λ is the user-defined parameter that controls the weights of the two terms.

### 3.5. Optimization

With the above objective, the back-propagation process can be derived. For the distillation objective, the gradient of the output is first computed as follows:

(20) $$\delta F_{i}^{D^{\prime }}=\frac{\partial L_{D}}{\partial F_{i}^{D}}=\exp\left(F^{D^{\prime }}⊙F^{D}/T\right)⊙F^{D^{\prime }}$$

From $\delta F_{i}^{D^{\prime }}$, the gradient of $F_{i}^{s^{\prime }}$ can be easily derived as follows:

(21) $$\begin{array}{cc} \delta F_{i}^{s^{\prime }}= & F_{i}^{D}F_{i}^{t}\Delta Softmax\left(F_{i}^{s^{\prime }}⊙F_{i}^{t}\right)\\ - & Softmax(F_{i}^{s^{\prime }}⊙F_{i}^{t}) \end{array}$$

where ΔSoftmax(·) is the gradient of the Softmax activation function. Then, the gradient $\delta F_{i}^{s^{\prime }}$ back-propagated through the projection operators and $\delta F^{s}$ is obtained, along with the back-propagated gradient from the tracking head.

After training, the test process follows CenterTrack, which maintains high tracking efficiency. Meanwhile, with the object feature distillation, the network inherits the detection performance of an accurate detection network. Moreover, the proposed distillation objective function focuses more on targets and, thus, is able to reduce the negative effects of feature distillation.

### 3.6. Tracklet Generation

For each input video frame, we output the center positions of all predicted targets and the prediction biases of the center points versus those in the previous frame. We then match the detected positions and the trajectories that are closest to those positions. At the same time, considering the small target position changes between adjacent frames, we limit the matching distance via the geometric mean of the length and width of the prediction boxes to avoid false matching over long periods. However, there is still a problem in that the prediction accuracy of targets with different sizes, moving speeds, and directions cannot be guaranteed with only one bias prediction head. For example, large prediction biases can be obtained for slow-moving objects with high frame rates or stationary targets, which are far from the real trajectories. As a consequence, we further adopt a simple and effective intersection over union (IOU)-based secondary matching method. Unmatched targets and trajectories from the first matching process are matched again, only according to the IOU scores between them. Meanwhile, to avoid false matches over long periods, we limit the successfully matched targets and trajectories that achieve IOU scores over 0.5. For trajectory initialization and updates, we use the settings in CenterTrack.

In addition, even with pixel-wise transformer distillation and temperature-controllable distillation, the distilled knowledge cannot be guaranteed to be completely correct. For example, due to the complex backgrounds of satellite videos, many background objects with similar appearances to targets could be wrongly recognized, which increases the number of false detections. Fortunately, due to the low confidence of these false detections, we found that they were usually temporally detected in one or two frames. As a consequence, we designed a 3-frame-based decision strategy. Different from DeepSORT, which completely abandons mismatched detections in two contiguous frames regardless of possible successful matches in subsequent frames, we defined a list of three components that records the matching status in three consecutive frames for each trajectory. If the detected targets successfully match the trajectories over three consecutive frames, then the three matches are determined and output. Otherwise, the targets are taken as false detections and abandoned. This strategy effectively reduces the influence of false detections on precision. Moreover, we remove trajectories that are unmatched over 30 consecutive frames and do not consider them as potential trajectories in subsequent frames.

## 4. Experimental Study

### 4.1. Dataset Description

Experiments were conducted by comparing our proposed method to a baseline and other current state-of-the-art MOT methods on three new and publicly available datasets, including AIR-MOT [10], SAT-MTB [48], and VISO [31].

The AIR-MOT [10] dataset is composed of 59 satellite videos, acquired by the Jilin-1 satellite. They dynamically recorded scenes from the airports and harbors in the cities of Abu Dhabi, Dubai, San Diego, Los Angeles, Sydney, Yokohama, Beijing, Sanya, and Shanghai from October 2017 to October 2020. Therefore, the annotated targets include aircraft and ships. In our experiments, we evaluated the methods separately on different targets, which were divided into two subsets, named AIR-aircraft and AIR-ship. Regarding the videos in the dataset, the size of the frames is 1920×1080 pixels and the spatial resolution ranges from 0.91 m to 1.27 m. The frame rate of the videos is 5–10 frames-per-second (FPS). In this dataset, aircraft and ships have different properties. Aircraft are usually large, sparsely arranged, and have distinguishable features, while ships are much smaller and densely arranged.

The videos in the SAT-MTB [48] dataset were also acquired by Jilin-1 but covered much larger regions across the USA, Mexico, Australia, Spain, Italy, France, Turkey, Japan, India, Thailand, Saudi Arabia, the United Arab Emirates, and China, with a spatial resolution of 0.92 m. The acquisition time was from 2017 to 2021, and there are 249 videos containing annotated targets in this dataset. The frame sizes of these videos range from 512×512 to 1080×2160 pixels, and the frame rate is about 10 FPS. The targets in this dataset were also divided into two subsets, named SAT-MTB-airplane and SAT-MTB-ship.

**Figure 3:**
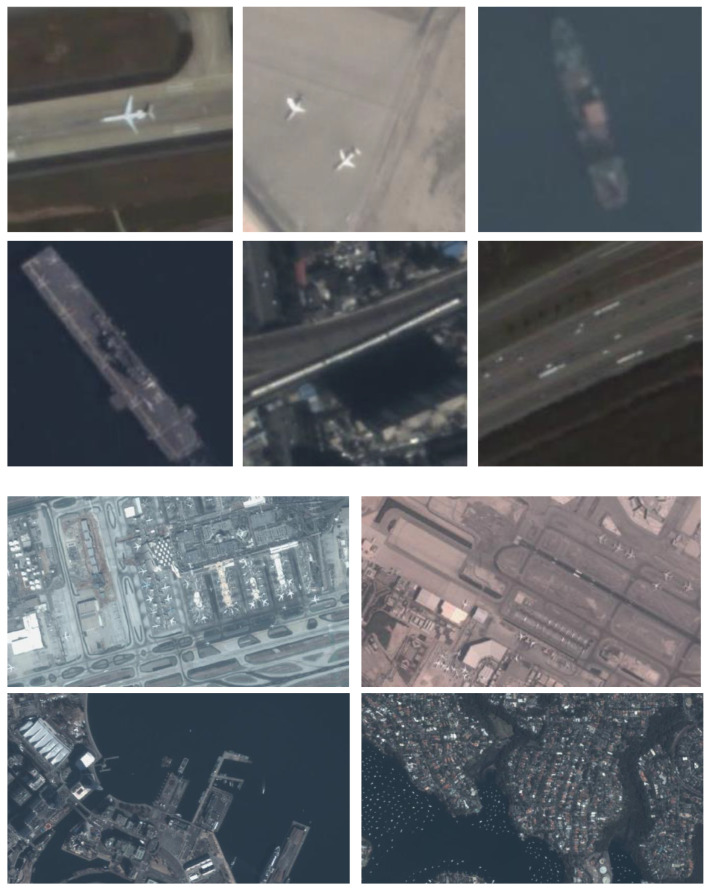
Some targets and images from the three datasets.

The VISO [31] dataset contains 47 videos that include annotated targets. They were also acquired by the Jilin-1 satellite, with a spatial resolution of 0.91 m. The frame sizes of these videos range from 1024×1024 to 1025×1025 and the frame rate is about 10 FPS. The targets in this dataset are mainly cars, which are also small and densely arranged. Some targets and scenes from the three datasets are illustrated in Figure 3.

For the division of training and test sets, we followed the standard training and test ratios of 1:1, 3:2, and 3:7 for the AIR-MOT, SAT-MTB, and VISO datasets, respectively, for fair comparison. As shown in the figures, the targets in these satellite videos were usually small, and the smallest size of detectable objects was 20 × 21 pixels.

### 4.2. Evaluation Metrics

We used fragmentation (FM), false positives (FPs), false negatives (FNs), mostly lost (ML), mostly tracked (MT), identification recall (IDR), identification precision (IDP), ID switches (IDs), identification F-score (IDF1), and multi-object tracking accuracy (MOTA) as the evaluation criteria, as do many satellite video MOT methods [10]. Among them, MOTA is a comprehensive evaluation metric that measures the overall accuracy of tracking as follows:

(22) $$\mathrm{MOTA}=1-\frac{\sum_{t}\left(\mathrm{FP}+\mathrm{FN}+\mathrm{IDs}\right)}{\sum_{t}\mathrm{GT}}$$

where *t* denotes the frame index and GT denotes the number of ground-truth bounding boxes in each frame. Larger MOTA values mean higher tracking accuracy, with 1 as the best case. IDF1 denotes the associated ratio among detected and tracked objects and is calculated as follows:

(23) $$\mathrm{IDF}1=\frac{2\mathrm{IDTP}}{2\mathrm{IDTP}+\mathrm{IDFP}+\mathrm{IDFN}}$$

where IDTP and IDFP represent the number of true IDs and false positive IDs, respectively, and IDFN is the number of false negative IDs. IDF1 stands for identification F-Scorel, and larger IDF1 values indicate more accurate associations. IDP and IDR denote the precision and recall, respectively, of ID tracking. MT and ML represent the ratio of correctly tracked trajectories in over 80% and below 20% of all frames, respectively. FM denotes the cut-off time of a trajectory. IDs represents the number of identification switches.

### 4.3. Implementation Details

Without loss of generality, in our experiments, we chose YOLOX [49] as the highly precise object detection network that was used to guide the tracking network, i.e., the baseline (CenterTrack), for feature distillation learning. In order to avoid errors caused by feature map scale mismatches during feature learning, for both networks, we resized the input images during training to 640×640. In addition, since our knowledge distillation module could be ignored during testing, we resized the images to 1088×608 for higher accuracy, which was more similar to the original scale of the images. Following CenterTrack’s original settings, we trained the model using the Adam optimizer for 70 epochs, accompanied by an initial learning rate of 1.25 × $10^{-4}$ and scaled down at 60 epochs by 10. We set the temperature to T=50 and the distillation weights λ to 0.2 and 0.4 for AIR-ship and AIR-aircraft targets, respectively. Considering that the partial AIR-ship dataset had an average of more than 100 targets per frame, we modified the maximum number of targets from 100 to 150 for this dataset. The other model parameters followed CenterTrack’s default settings, and more experimental details can be found in CenterTrack.

### 4.4. Evaluation of λ

**Figure 4:**
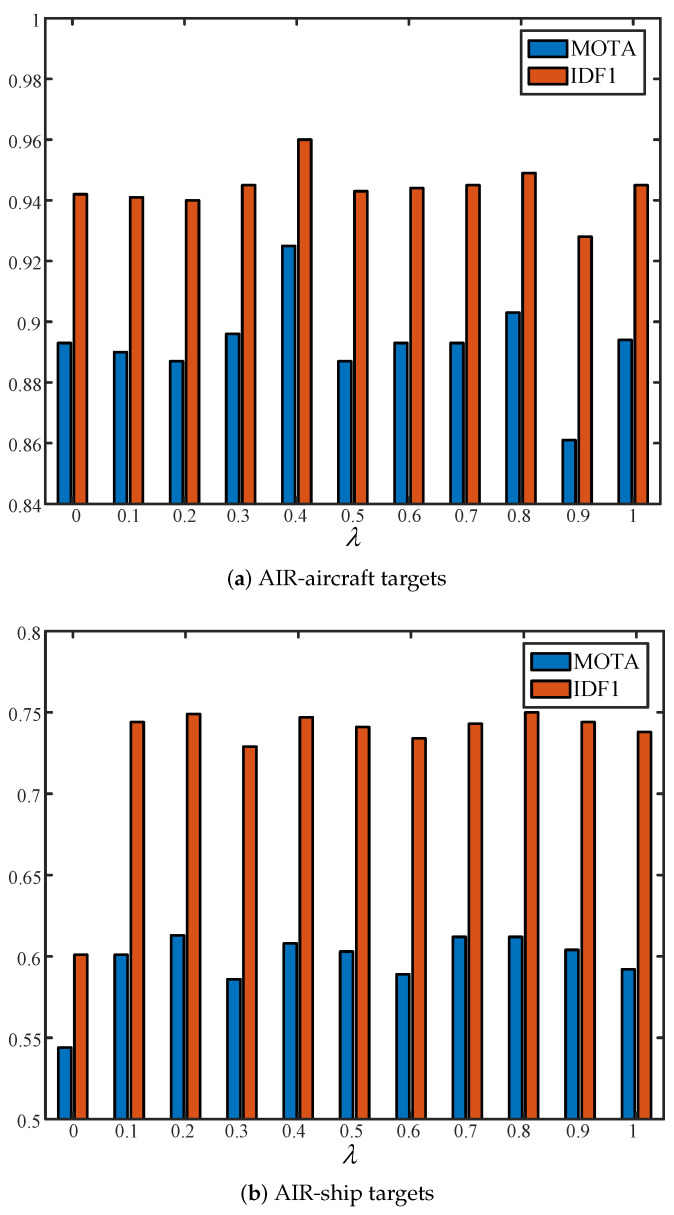
Evaluation of λ on AIR-ship and AIR-aircraft targets.

Since the characteristics of airplanes and ships are different in the AIR-MOT dataset, we tested the most suitable λ on two partial datasets separately. We selected the two most important indicators out of the multi-objective tracking evaluation metrics, namely, MOTA and IDF1, as shown in Figure 4. Figure 4a shows the results for the AIR-aircraft dataset and Figure 4b shows the results for the AIR-ship dataset.

As shown in Figure 4, the most suitable λ was 0.2. When the value of λ was 0.2, the MOTA value reached 0.6140 and the IDF1 value reached 0.7430. Therefore, in the subsequent experiments, the λ for the AIR-aircraft dataset was set to 0.2. As shown in Figure 4, when λ was 0.4, the MOTA value reached 0.9270 and the IDF1 value reached 0.9610. Therefore, for the subsequent experiments, the λ for the AIR-ship dataset was set to 0.4.

### 4.5. Ablation Studies

**Figure 5:**
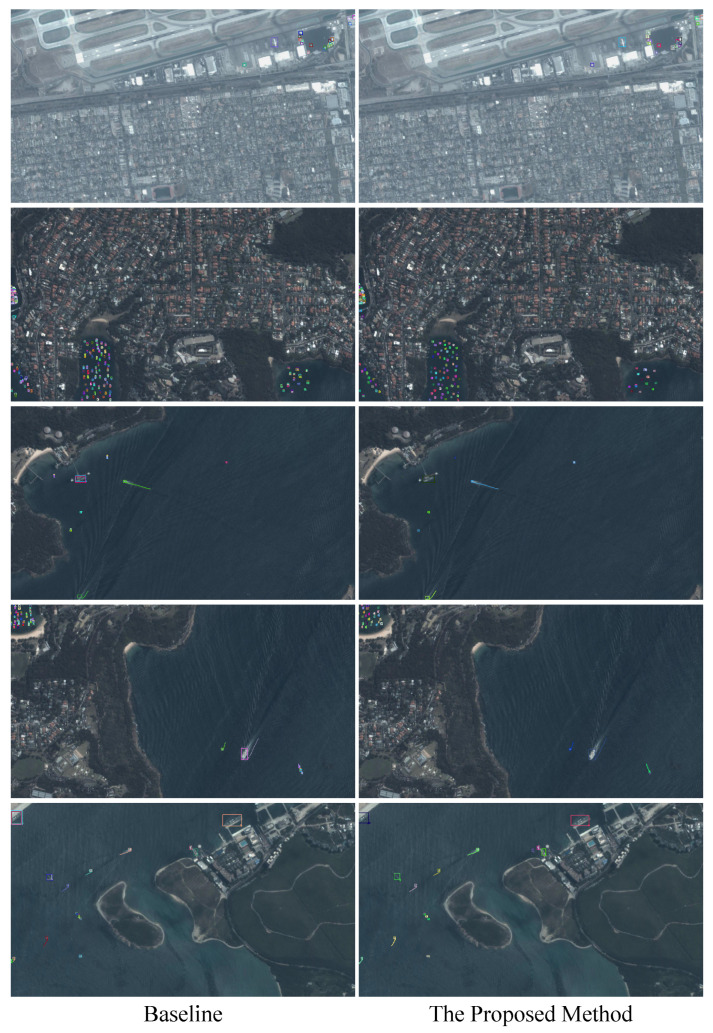
Visualization of several tracking results using the baseline method (CenterTrack) and the proposed method.

We selected CenterTrack as the baseline method to conduct ablation experiments on the AIR-aircraft and AIR-ship datasets. The visualized results are illustrated in Figure 5. The quantified comparison results are shown in Table 2 and Table 3. The “Tracklets” label represents the tracklet generation approach designed in this study, and the “Transformer” label refers to the transformer distillation module proposed in this paper. As can be seen from Table 2, the proposed method exceeded the baseline MOTA by about 7%. In terms of IDF1, the proposed method exceeded the baseline by 14.2%. In addition, in terms of FM, ML, MT, IDR, IDP, and IDs, the proposed method improved on the baseline by 85, 8, 14, 13.8%, 16.9%, and 2411, respectively. This demonstrates that the proposed approach is obviously superior to the baseline method.

As can be seen from Table 3, in terms of MOTA, the proposed method was 3.30% higher than the baseline. For FM, ML, MT, IDR, IDP, IDs, and IDF1, the proposed method improved on the baseline by 6, 2, 5, 1.4%, 1.70%, 5, and 1.90%, respectively. This further demonstrates the effectiveness of the proposed method on the AIR-aircraft dataset. On the one hand, transformer-based feature distillation increases the accuracy of object detection, which in turn improves multi-target tracking performance. On the other hand, the pixel-wise distillation method also improves performance.

### 4.6. Comparison to State-of-the-Art MOT Methods

#### 4.6.1. Evaluation on the AIR-MOT Dataset

In order to evaluate the performance of the proposed method, we selected both DBT-based and JDT-based methods for comparison. Most of these methods are recently proposed, popular, and state-of-the-art methods. The DBT-based methods included StrongSORT [15], SORT [11], DeepSORT [13], GHOST [28], CKDNet-SMTNet [32], and DSFNet [33]. The JDT-based methods included FairMOT [21], TraDeS [22], SGT [50], UMA [17], CenterTrack [19], and TGraM [10]. In addition, we also selected transformer-based methods, including TrackFormer [30] and MOTR [38].

Table 4 shows the experimental results for aircraft and ship targets on the AIR-MOT dataset. From Table 4, we can see that the proposed method exceeded the second-ranked TGraM by 6.4% in terms of MOTA for aircraft targets. This demonstrates the superior performance of our method. For ship targets, the results showed that the proposed method exceeded the second-ranked method by 6.7% in terms of MOTA. The ship targets were usually small and closely arranged, which demonstrates that our method performs well on densely arranged, smaller targets. In addition, when the basic performance for aircraft targets was very high, our method still showed a significant improvement. This further verifies the superior performance of the proposed method.

#### 4.6.2. Evaluation on the SAT-MTB Dataset

To verify the generalization performance of our method, we selected both DBT-based and JDT-based methods for comparison on another recently proposed remote sensing dataset, namely SAT-MTB [48]. The DBT-based methods included ByteTrack [12], DeepSORT [13], Trackor++ [51], CKDNet-SMTNet [32], DeepSORT [13], and Trackor++ [51]. The JDT-based methods included FairMOT [21] and TGraM [10].

Table 5 shows the experimental results for ship and airplane targets on the SAT-MTB dataset. In Table 5, it can be seen that the proposed method exceeded the second-ranked CenterTrack by 3.8% in terms of MOTA for ship targets. This demonstrates the state-of-the-art performance of the proposed method on the SAT-MTB database. From Table 5, we can also see that the proposed method exceeded the second-ranked method by 7.7% in terms of MOTA for airplane targets. Since ship targets were usually small and closely arranged, the results showed that the proposed method had the highest performance, which demonstrates that our method performs well on densely arranged, small targets. In addition, the basic tracking performance of airplane targets was already high, but the proposed method still exceeded the second-ranked method by 7.7% in terms of MOTA, further verifying the generalization performance of our method.

#### 4.6.3. Evaluation on the VISO Dataset

We also verified the performance of our method on the VISO [31] dataset. The methods selected for comparison included MMB+FairMOT [31], DSFNet [33], MMB+CMOT [31], CKDNet-SMTNet [32], DSFNet+SORT [33], MMB+DTTP [31], and CenterTrack [19].

Table 6 shows the experimental results for car targets on the VISO dataset. From Table 6, it can be seen that the proposed method exceeded the second-ranked CenterTrack [19] by 6.1% in terms of MOTA. The proposed method also had the best performance in terms of IDF1 and IDR. This not only verifies the superior performance of our method on the VISO dataset but also verifies the generalization performance of our method.

**Figure 6:**
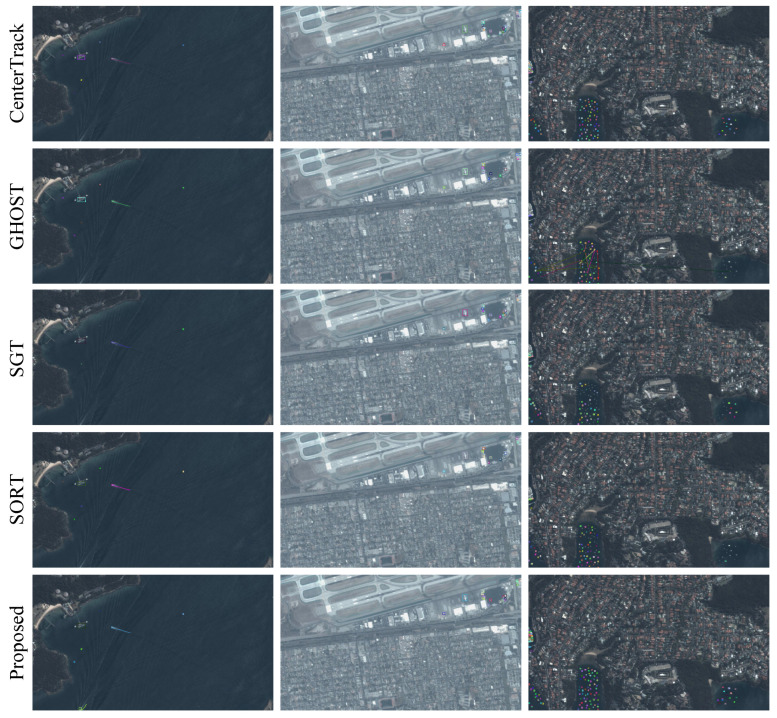

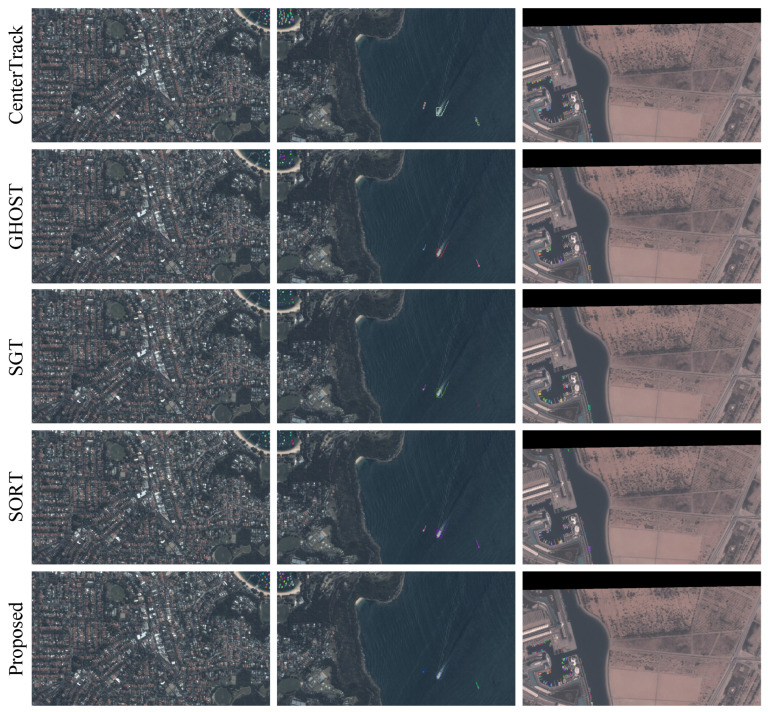
Visualization of several tracking results using the baseline method (CenterTrack) and the proposed method.

In addition to quantitative comparisons, we also visualized experimental results on several sequences, as shown in Figure 6. To save space, we only present visualizations of the top three methods. As can be seen in Figure 6, our method performed the best in terms of ID continuity, missed detections, and false detections. This also demonstrates that the use of pixel-wise transformer distillation and temperature-controllable key feature learning is obviously superior to existing methods for remote sensing targets.

## 5. Conclusions

MOT in satellite videos is significant in many important applications. Due to the limitations of satellite computing devices and the need for high tracking performance for many crucial applications, designing efficient and accurate tracking frameworks is imperative. As a consequence, in this paper, we propose a new transformer distillation-based end-to-end joint detection and tracking method. In this method, using a highly precise teacher network, object features are distilled into a JDT method to improve localization and tracking accuracy while maintaining tracking efficiency. Considering the scale variance between different objects, such as ships and aircraft, we propose a pixel-wise transformer distillation module to effectively distill useful target features. Moreover, a temperature-controllable feature distillation objective function is also proposed to distill critical features. Experiments on aircraft, ship, and car targets demonstrated the significant improvements offered by the proposed method over the baseline and current state-of-the-art methods. The proposed method achieved an overall tracking performance of over 90% on the AIR-MOT dataset. The proposed method inherits advantages from both DBT- and JDT-based methods during tracking. However, it reduces training efficiency with object detection network and distillation learning. In future work, we will try to use existing large remote sensing models to facilitate the training of the tracking network.

## Figures and Tables

**Table 1:** Comparison between DBT and JDT methods.

	Flowchart	Properties
DBT	Object detection + data association	High detection accuracy; Low tracking efficiency
JDT	End-to-end	Feature sharing; High tracking efficiency; Low stability
Proposed	End-to-end + distillation	Feature sharing; High tracking efficiency; High detection accuracy

**Table 2:** Results of ablation experiments on AIR-ship targets.

Method	FM	ML	MT	IDR	IDP	IDs	IDF1	MOTA
Baseline	640	143	297	52.40%	70.30%	2461	60.10%	54.40%
Baseline + Tracklets	638	141	292	62.65%	84.40%	71	72.00%	56.90%
Baseline + Transformer	701	143	291	56.90%	79.50%	234	67.30%	59.39%
Proposed Method	555	135	311	66.10%	87.20%	50	74.30%	61.40%

**Table 3:** Results of ablation experiments on AIR-aircraft targets.

Method	FM	ML	MT	IDR	IDP	IDs	IDF1	MOTA
Baseline	35	9	184	93.70%	94.80%	12	94.20%	89.30%
Baseline + Tracklets	41	9	182	93.70%	94.90%	5	94.10%	89.60%
Baseline + Transformer	31	5	183	95.10%	96.60%	5	95.90%	92.60%
Proposed Method	29	7	189	95.10%	96.50%	7	96.10%	92.70%

**Table 4:** Comparison of results from the proposed method and several state-of-the-art methods on the AIR-MOT dataset.

AIR-aircraft								targets
Method	FM	ML	MT	IDR	IDP	IDs	IDF1	MOTA
SORT [11]	16	17	183	90.0%	99.5%	7	94.5%	89.7%
UMA [17]	81	11	186	91.4%	92.8%	19	92.1%	85.4%
CenterTrack [19]	35	9	184	93.7%	94.8%	12	94.2%	89.3%
TraDeS [22]	114	44	148	75.0%	97.4%	42	84.7%	73.6%
FairMOT [21]	226	8	180	92.2%	94.2%	23	93.2%	86.7%
CKDNet-SMTNet [32]	52	3	187	95.7%	85.0%	36	90.0%	82.1%
TrackFormer [30]	110	12	184	90.5%	87.1%	97	88.8%	80.2%
TGraM [10]	181	8	174	90.7%	94.1%	28	92.4%	86.3%
StrongSORT [15]	14	18	182	88.2%	99.6%	1	93.5%	87.8%
SGT [50]	153	6	184	93.9%	94.6%	56	94.3%	89.2%
GHOST [28]	67	15	183	90.1%	98.8%	2	94.3%	89.6%
DSFNet-SORT [33]	96	28	164	83.1%	98.9%	6	90.3%	82.3%
Proposed Method	29	7	189	95.1%	96.5%	7	96.1%	92.7%
AIR-ship targets								
Method	FM	ML	MT	IDR	IDP	IDs	IDF1	MOTA
SORT [11]	434	254	236	49.7%	92.0%	80	64.5%	47.5%
CenterTrack [19]	640	143	297	52.4%	70.3%	2461	60.1%	54.4%
UMA [17]	1193	204	277	56.2%	71.6%	250	62.9%	39.8%
CKDNet-SMTNet [32]	1375	238	175	40.1%	59.5%	516	47.9%	25.0%
FairMOT [21]	2487	262	187	43.3%	77.4%	258	55.5%	34.7%
TraDeS [22]	1265	261	202	47.9%	75.7%	118	58.6%	35.0%
TGraM [10]	1449	311	124	31.7%	71.7%	166	43.9%	25.1%
TrackFormer [30]	1439	173	286	55.6%	62.7%	1360	58.9%	34.9%
StrongSORT [15]	311	312	206	42.4%	95.9%	19	58.8%	41.0%
SGT [50]	1718	238	217	47.4%	87.7%	717	61.5%	44.8%
DSFNet-SORT [33]	1045	352	135	33.2%	83.3%	36	47.5%	27.0%
GHOST [28]	927	246	239	50.5%	90.8%	162	64.9%	47.8%
Proposed Method	555	135	311	66.1%	87.2%	50	74.3%	61.4%

**Table 5:** Comparison of results from the proposed method and several state-of-the-art methods on the SAT-MTB dataset.

SAT-MTB-ship targets								
Method	FM	ML	MT	IDR	IDP	IDs	IDF1	MOTA
DeepSORT [13]	-	40	35	-	-	144	45.5%	13.5%
Trackor++ [51]	-	30	35	-	-	119	56.6%	34.3%
CenterTrack [19]	325	13	39	68.5%	69.4%	84	68.3%	54.1%
FairMOT [21]	-	39	23	-	-	143	45.0%	8.2%
CKDNet-SMTNet [32]	-	14	43	-	-	299	49.8%	36.9%
TGraM [10]	-	47	19	-	-	283	35.6%	−13.9%
ByteTrack [12]	-	34	38	-	-	168	46.9%	10.5%
DSFNet [33]	-	16	38	-	-	235	57.7%	37.2%
Proposed Method	313	11	45	69.2%	72.0%	82	71.6%	57.9%
SAT-MTB-airplane targets								
Method	FM	ML	MT	IDR	IDP	IDs	IDF1	MOTA
DeepSORT [13]	-	33	22	-	-	0	55.3%	35.6%
Trackor++ [51]	-	13	33	-	-	64	57.8%	17.6%
CenterTrack [19]	38	8	45	76.2%	77.7%	38	76.9%	56.7%
FairMOT [21]	-	22	32	-	-	1	63.9%	32.0%
CKDNet-SMTNet [32]	-	3	53	-	-	91	65.9%	54.3%
TGraM [10]	-	18	33	-	-	6	67.5%	37.4%
ByteTrack [12]	-	26	31	-	-	1	65.1%	45.3%
Proposed Method	36	7	47	74.1%	82.3%	66	77.0%	64.4%

**Table 6:** Comparison of results from the proposed method and several state-of-the-art methods on the VISO dataset (car targets).

Method	FM	ML	MT	IDR	IDP	IDs	IDF1	MOTA
CenterTrack [19]	3086	328	275	34.6%	67.3%	5503	45.7%	45.3%
CKDNet-SMTNet [32]	2887	443	105	22.6%	62.8%	3071	33.2%	33.5%
MMB+FairMOT [31]	205	623	21	-	-	52	-	2.3%
DSFNet [33]	1326	432	45	-	-	348	-	3.6%
MMB+CMOT [31]	111	494	38	-	-	89	-	22.8%
DSFNet+SORT [33]	8075	105	318	-	-	434	-	41.6%
MMB+DTTP [31]	1344	22	483	-	-	3090	-	44.5%
Proposed Method	3019	277	355	40.1%	67.4%	5810	49.3%	51.4%

## Data Availability

Data are unavailable due to privacy.
